# Sarcopenia as a Determinant of Blood Pressure in Older Koreans: Findings from the Korea National Health and Nutrition Examination Surveys (KNHANES) 2008–2010

**DOI:** 10.1371/journal.pone.0086902

**Published:** 2014-01-29

**Authors:** Kyungdo Han, Yu-Mi Park, Hyuk-Sang Kwon, Seung-Hyun Ko, Seung-Hwan Lee, Hyeon Woo Yim, Won-Chul Lee, Yong Gyu Park, Mee Kyoung Kim, Yong-Moon Park

**Affiliations:** 1 Department of Preventive Medicine, The Catholic University of Korea, Seoul, Korea; 2 Department of Biostatistics, The Catholic University of Korea, Seoul, Korea; 3 School of Medicine, The Catholic University of Korea, Seoul, Korea; 4 Division of Endocrinology and Metabolism, Department of Internal Medicine, The Catholic University of Korea, Seoul, Korea; 5 Department of Epidemiology and Biostatistics, Arnold School of Public Health, University of South Carolina, Columbia, South Carolina, United States of America; Shanghai Institute of Hypertension, China

## Abstract

**Background:**

Blood pressure (BP) is directly and causally associated with body size in the general population. Whether muscle mass is an important factor that determines BP remains unclear.

**Objective:**

To investigate whether sarcopenia is associated with hypertension in older Koreans.

**Participants:**

We surveyed 2,099 males and 2,747 females aged 60 years or older.

**Measurements:**

Sarcopenia was defined as an appendicular skeletal muscle mass divided by body weight (ASM/Wt) that was <1 SD below the gender-specific mean for young adults. Obesity was defined as a body mass index (BMI) ≥25 kg/m^2^. Subjects were divided into four groups based on presence or absence of obesity or sarcopenia. Hypertension was defined as a systolic BP (SBP) ≥140 mmHg, a diastolic BP (DBP) ≥90 mmHg, or a self-reported current use of antihypertensive medications.

**Results:**

The overall prevalence of hypertension in the four groups was as follows 49.7% for non-obese non-sarcopenia, 60.9% for non-obese sarcopenia, 66.2% for obese non-sarcopenia and 74.7% for obese sarcopenia. After adjustment for age, gender, regular activity, current smoking and alcohol use, the odds ratio (OR) for having hypertension was 1.5 (95% confidence interval (CI) = 1.23–1.84) in subjects in the non-obese sarcopenia group, 2.08 (95% CI = 1.68–2.57) in the obese non-sarcopenia group and 3.0 (95% CI = 2.48–3.63) in the obese sarcopenia group, compared with the non-obese non-sarcopenia group (*p* for trend <0.001). Controlling further for body weight and waist circumference did not change the association between hypertension and sarcopenia. The association between sarcopenia and hypertension was more robust in the subjects with diabetes mellitus.

**Conclusion:**

Body composition beyond BMI has a considerable impact on hypertension in elderly Koreans. Subjects with sarcopenic obesity appear to have a greater risk of hypertension than simply obese or sarcopenia subjects.

## Introduction

Sarcopenia, the age-associated loss of muscle mass, is related to deteriorations in physical disability, metabolic impairments, and increased mortality [1]. In a study including 1,396 men and women aged 70 years and older, low arm muscle area was associated with an elevated mortality rate during an 8-year follow-up period [2]. Low muscle mass has been associated with cardiovascular risk factors including arterial stiffness [3], suggesting the additive effects of low muscle mass on blood pressure (BP). With aging, lean body mass decreases, whereas fat mass increases. Recently, Lim et al. found that sarcopenic obesity, defined by appendicular muscle mass/body weight (ASM/Wt), was more closely associated with metabolic syndrome than either sarcopenia or obesity alone [4]. Therefore, sarcopenia and obesity might act synergistically on metabolic and functional impairments in the elderly.

BP is directly and causally associated with body size in the general population. Total body mass contains two factors that have opposite biological effects, namely adipose tissue and lean mass. Whereas adipose tissue has been associated with deleterious health outcomes, preserved lean mass is positively associated with physical fitness, higher caloric expenditure and exercise capacity, all of which are associated with better survival [5]. Body fat stores, per se, appear to have no impact on BP or the risk of hypertension after adjustment for the associated increases in resting energy expenditure [6], [7]. However, it remains unclear whether body composition (especially, muscle mass) is an important factor that determines BP.

In Korea, hypertension is the most important cause of CVD [8]. The Korean population differs importantly in CVD patterns compared with Caucasians [9]. The major causes of death in Western countries are atherosclerotic coronary diseases. In contrast, in Korea, hypertensive heart disease and stroke are more common [8], [10]. Therefore, we examined the relationship between sarcopenic obesity and hypertension, which is the major risk factor for CVD in the elderly population, particularly in Korea.

## Materials and Methods

### Ethic Statement

The study protocol was reviewed and approved by the Korea Centers for Disease Control and Prevention (KCDC) institutional review board (2008-04EXP-01-C, 2009-01CON-03-2C, 2010-02CON-21-C), and written consent was obtained from the subjects.

### Subjects

This cross-sectional study was based on data acquired in the Fourth and Fifth Korea National Health and Nutrition Examination Survey (KNHANES IV-V) conducted from 2008 to 2010. These surveys have been conducted periodically since 1998 to assess the health and nutritional status of the non-institutionalized civilian population in Korea [11], [12]. The KNHANES is composed of a health interview survey, a health examination survey, and a nutrition survey conducted by trained investigators. The KNHANES employed a rolling sampling design that implemented a complex, stratified, multistage probability-cluster survey of a representative Korean population sample. Additional details regarding the study design and methods are provided elsewhere [11], [12]. The total number of participants in this analysis was 4,846 (2,099 males and 2,747 females) subjects aged 60 years or older among those who participated in the survey between January 2008 and December 2010. We excluded participants who had any malignancy, missing data for variables included in the analysis, or were pregnant.

### Definition of Sarcopenia and Obesity

ASM was measured by DXA (QDR 4500A, Hologic Inc., Waltham, MA, USA). ASM (kg) was defined as the sum of the lean soft tissue masses of the arms and legs, after the method of Heymsfield *et al*., [13]. We used ASM as a percentage of body weight (ASM/Wt), as modified from the study of Janssen *et al.,* [14]. Sarcopenia was defined [4] as an ASM/Wt <1 SD below the mean of a sample of healthy adults aged 20 to 40 years. For males, the cutoff value for sarcopenia was 30.5% (ASM/Wt), defined as less than 1 SD below the gender-specific normal mean for the young reference group. For females, the corresponding limit was 23.9% (ASM/Wt). A subject was classified as obese if his or her BMI was ≥ 25 kg/m^2^ [15]. We first classified the subjects as obese or non-obese. The subjects were further classified into non-obese non-sarcopenia, non-obese sarcopenia, obese non-sarcopenia, or obese sarcopenia groups according to the definitions above.

### Definition of Hypertension

BP was measured three times on the right arm while the individual was in a seated position after at least 5 min of rest using a mercury sphygmomanometer (Baumanometer; Baum, Copiague, NY, USA). The final BP value was obtained by averaging the values of the second and third measurements. We recorded the blood pressure to the closest 2 mm Hg on the manometer. Table S1 in File S1 shows the distribution of the last digits recorded for systolic and diastolic blood pressure values, the preference for zero as the last digit in systolic blood pressure values in 54 and 38%, and in diastolic blood pressure values in 38 and 45% of the measurements. Hypertension was defined as an SBP≥140 mmHg, a DBP≥90 mmHg, or a self-reported current use of antihypertensive medications. We classified the BP into four stages according to severity [16]; Normal (Level 1), SBP<120 mmHg and DBP<80 mmHg; Prehypertension (Level 2), 120≤ SBP<140 mmHg or 80≤ DBP<90 mmHg; Mild hypertension (Level 3), 140≤ SBP<160 mmHg or 90≤ DBP<100 mmHg; and Moderate or Severe hypertension (Level 4), SBP≥160 mmHg or DBP≥100 mmHg.

### Statistical Analysis

All data are presented as means ± standard error (SE) unless stated otherwise. If necessary, logarithmic transformation was performed to achieve a normal distribution. The participants’ characteristics were compared according to sarcopenia status using independent-sample Student’s *t*-tests for continuous measures and χ^2^ tests for categorical measures. Pearson’s correlation coefficients between BP and various parameters of body composition were calculated. We tested whether hypertension (or systolic/diastolic blood pressure) could be explained by the interaction between sarcopenia and obesity by two-way ANCOVA with adjustment for age, sex, regular activity, current smoking, alcohol use, previous CHD, stroke, diabetes mellitus, and dyslipidemia as covariates. The effect of the interaction between sarcopenia and obesity on hypertension (or systolic/diastolic blood pressure) was not significant (P = 0.629). Therefore, the interaction term was not included in subsequent analyses.

Multiple linear regression analysis and multiple logistic regression analysis were performed to examine the independent effect of ASM (kg) on BP levels and hypertension, respectively.

Multiple logistic regression analyses were also used to assess the associations between the four body composition categories and hypertension. In addition, tests for linear trends of prevalence or proportion across each group were conducted using a logistic regression analysis. To explore whether a particular BMI cutoff value for obesity would affect the associations, we used a BMI of 28 kg/m^2^ as a threshold to define obesity in the sensitivity analysis. In addition, subgroup analyses were conducted according to sex and the presence of diabetes mellitus. Statistical analyses were performed using the survey procedure of the SAS software (version 9.2; SAS Institute, Cary, NC, USA) to account for the complex sampling design and to provide nationally representative prevalence estimates. P<0.05 was accepted as indicative of significance.

## Results

### General Characteristics of the Study Population

The cross-sectional analyses included data of 4,846 participants (2,099 males and 2,747 females). The mean age of the study participants was 69.5 years. In both the obese and non-obese groups, subjects with sarcopenia were older and were less likely to exercise regularly and were less likely to have an occupation, more likely to live in an urban area, compared with subjects without sarcopenia (Table 1). Subjects with sarcopenia had higher triglyceride levels, increased total white blood cell (WBC) counts and greater insulin resistance (high HOMA-IR). There was significant difference in SBP levels between non-obese non-sarcopenia and non-obese sarcopenia group (P = 0.002). However, there was no significant difference in systolic or diastolic BP levels between obese non-sarcopenia and obese sarcopenia. In both the obese and non-obese groups, subjects with sarcopenia were more likely to have previous hypertension and take antihypertensive medications, compared with those without sarcopenia (Table 1).

**Table 1:** Clinical characteristics of study population.

	Non-obese			Obese		
	Sarcopenia (−)	Sarcopenia (+)	P-Value	Sarcopenia (−)	Sarcopenia (+)	P-Value
	(n = 2,326)	(n = 894)		(n = 594)	(n = 1,032)	
SBP (mmHg)	126.4±0.5	129.3±0.9	0.002	129.9±0.8	131.2±0.7	0.230
DBP (mmHg)	74.3±0.3	75.1±0.5	0.140	76.9±0.5	77.6±0.4	0.278
Previous Hypertension (%)	35.5 (1.3)	47.7 (2.0)	<0.001	56.0 (2.4)	64.4 (1.8)	<0.001
Subjects taking antihypertensive medications (%)	33.0 (1.3)	45.3 (1.9)	<0.001	52.0 (2.4)	62.3 (1.9)	0.001
Age (yrs)	69.8±0.2	70.6±0.3	0.020	67.3±0.3	69.1±0.3	<0.001
Sex (Men)	44.8 (1.1)	51.3 (1.9)	0.0057	35.3 (2.3)	36.6 (1.8)	0.6544
BMI (kg/m^2^)	21.7±0.1	22.9±0.1	<0.001	26.6±0.1	27.6±0.1	<0.001
Waist Circumference (cm)	78.4±0.2	83.4±0.2	<0.001	90.0±0.3	93.1±0.3	<0.001
Total body fat percentage (%)	25.8±0.2	32.5±0.3	<0.001	31.2±0.3	37.0±0.3	<0.001
ASM (kg)	16.1±0.1	14.9±0.1	<0.001	18.5±0.2	16.6±0.1	<0.001
ASM/Wt (%)	29.6±0.1	25.8±0.1	<0.001	27.7±0.2	24.3±0.1	<0.001
Total cholesterol (mg/dL)	189.4±0.9	193.7±1.6	0.015	194.7±1.7	196.7±1.4	0.366
HDL-cholesterol (mg/dL)	51.2±0.3	49.0±0.5	0.001	47.5±0.6	48.0±0.5	0.522
Triglycerides (mg/dL)	132.4±2.4	151.9±3.7	<0.001	153.1±4.7	164.9±3.7	0.008
Fasting glucose (mg/dL)	100.6±0.6	107.0±1.2	<0.001	106.0±1.2	108.6±1.2	0.111
HOMA-IR	2.2±0.0	2.6±0.1	<0.001	3.0±0.1	3.4±0.1	<0.001
WBC count (10^3^/µL)	5.9±0.0	6.5±0.1	<0.001	5.9±0.1	6.4±0.1	<0.001
AST (IU/L)	24.4±0.3	24.2±0.6	0.252	24.6±0.5	25.2±0.6	0.632
ALT (IU/L)	19.2±0.3	21.0±0.5	0.001	23.5±0.7	23.6±0.6	0.991
Previous CHD (%)	3.4 (0.4)	7.0 (0.9)	<0.001	3.8 (1.0)	8.4 (1.0)	<0.001
Previous stroke (%)	2.4 (0.4)	4.3 (0.7)	0.001	1.3 (0.5)	4.1 (0.6)	0.001
Regular exercise a (%)	22.2 (1.2)	14.7 (1.5)	<0.001	27.9 (2.3)	19.8 (1.5)	<0.001
Regions, urban area (%)	62.9 (3.0)	71.8 (2.9)	<0.001	68.3 (3.4)	73.4 (2.9)	0.001
Occupation, yes (%)	43.2 (1.8)	30.0 (2.2)	<0.001	45.3 (2.9)	32.9 (1.7)	<0.001
Dietary intake						
Total energy (kcal/day)	1719.7±19.7	1585.3±28.3	<0.001	1822.3±40.4	1612.1±27.5	<0.001
Protein (% of energy)	13.0±0.1	13.4±0.2	0.018	13.3±0.2	13.6±0.2	0.132
Fat (% of energy)	11.5±0.2	12.4±0.3	0.009	12.0±0.4	13.0±0.3	0.028
Carbohydrate (% of energy)	75.5±0.3	74.2±0.4	0.005	74.7±0.5	73.4±0.3	0.025

Data are presented as mean ± SE or as % (SE).

SBP, systolic blood pressure; DBP, diastolic blood pressure; BMI, body mass index; ASM, appendicular skeletal muscle mass; HDL, high-density lipoprotein; HOMA-IR, homeostasis model assessment-insulin resistance; WBC, white blood cell; AST, aspartate aminotransferase; ALT, alanine aminotransferase; CHD, coronary heart disease.

a Regular physical activity was indicated as yes when the subject does severe exercise on a regular basis (for more than 20 min at a time and more than three times per week).

Pearson’s correlation analysis showed that SBP was correlated negatively with ASM (*r* = −0.068) and ASM/Wt. (*r* = −0.136) and positively with BMI (*r* = 0.197), WC (*r* = 0.101), total body fat mass (*r* = 0.119), and total body fat percentage (*r* = 0.127) (all *p*<0.001). DBP was correlated negatively with ASM/Wt. (*r* = −0.102) and positively with ASM (*r* = 0.080), BMI (*r* = 0.186), WC (*r* = 0.123), total body fat mass (*r* = 0.164), and total body fat percentage (*r* = 0.101) (all *p*<0.001; Table 2).

**Table 2:** Pearson’s correlation coefficients between blood pressure and anthropometric parameters.

SBP	*R*	P-Value
ASM/Wt.	−0.136	<0.001
ASM (kg)	−0.068	0.001
BMI (kg/m^2^)	0.197	<0.001
Waist circumference (cm)	0.101	<0.001
Total body fat mass (kg)	0.119	<0.001
Total body fat percentage (%)	0.127	<0.001
**DBP**	***R***	**P-Value**
ASM/Wt.	−0.102	<0.001
ASM (kg)	0.080	<0.001
BMI (kg/m^2^)	0.186	<0.001
Waist circumference (cm)	0.123	<0.001
Total body fat mass (kg)	0.164	<0.001
Total body fat percentage (%)	0.101	<0.001

### Prevalence of Hypertension According to the Four Body Composition Categories

**Figure 1:**
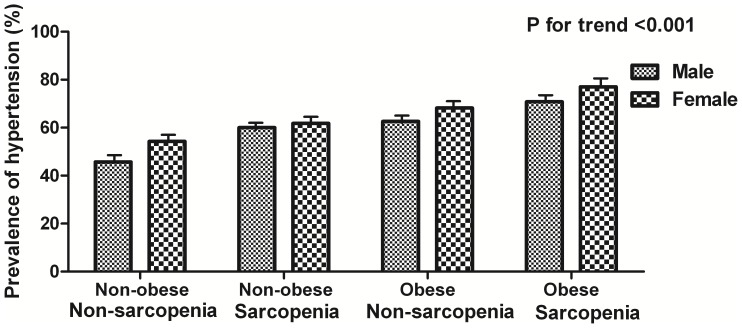
Prevalence of hypertension according to the 4 body composition categories based on obesity and sarcopenia.

Subjects were divided into four groups according to the definition of obesity and sarcopenia: non-obese non-sarcopenia, non-obese sarcopenia, obese non-sarcopenia and obese sarcopenia groups. The overall prevalence of hypertension in the four groups was as follows: 49.7% for non-obese non-sarcopenia, 60.9% for non-obese sarcopenia, 66.2% for obese non-sarcopenia and 74.7% for obese sarcopenia (*p* for trend <0.001; Figure 1). In both obese and non-obese groups, subjects with sarcopenia had a higher prevalence of hypertension than subjects without sarcopenia.

**Figure 2:**
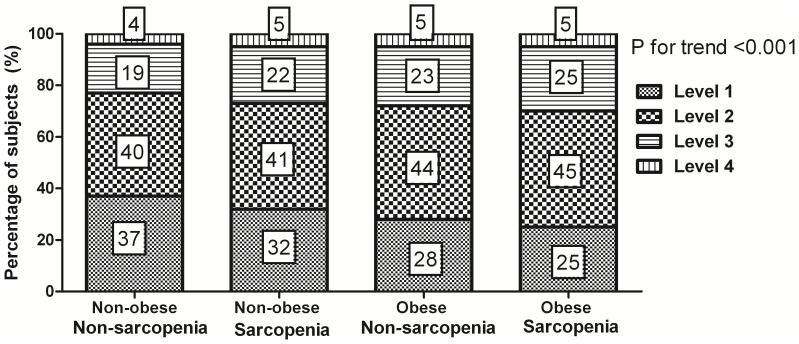
The distribution of blood pressure levels according to the 4 body composition categories. Subjects taking anti-hypertensive medications were excluded from this analysis. Level 1; SBP<120 mmHg and DBP<80 mmHg, Level 2; 120≤SBP<140 mmHg or 80≤DBP<90 mmHg, Level 3; 140≤SBP<160 mmHg or 90≤DBP<100 mmHg, Level 4; SBP≥160 mmHg or DBP≥100 mmHg.

We analyzed the distribution of BP levels according to these groups, excluding the subjects taking anti-hypertensive medications. The proportion of subjects with a SBP<120 mmHg and a DBP<80 mmHg decreased significantly from 37% in the non-obese non-sarcopenia group to 25% in the obese sarcopenia group (*p* for trend <0.001; Figure 2). The proportion of subjects with prehypertension (Level 2; 120≤ SBP<140 mmHg or 80≤ DBP<90 mmHg) increased significantly from 40% in the non-obese non-sarcopenia group to 45% in the obese sarcopenia group (*p* for trend <0.001). The proportion of subjects with hypertension (Level 3 and 4) increased linearly from 23% in the non-obese non-sarcopenia group to 30% in the obese sarcopenia group.

### Multivariable Analyses for the Associations between Hypertension and the Four Body Composition Categories

After adjustment for age, gender, and lifestyle factors (regular activity, current smoking and alcohol use), the OR for having hypertension was 1.5 (95% CI = 1.23–1.84) in subjects in the non-obese sarcopenia group, 2.08 (95% CI = 1.68–2.57) in the obese non-sarcopenia group and 3.0 (95% CI = 2.48–3.63) in the obese sarcopenia group, compared with the non-obese non-sarcopenia group (*p* for trend <0.001; Table 3). Controlling further for previous coronary heart disease, stroke history and metabolic risk factors (model 2) did not change the association between hypertension and obesity sarcopenia (OR = 2.52; 95% CI 2.06–3.09, *p* for trend <0.001). Further adjustment for body weight (model 3) reduced the magnitude of the ORs for hypertension, but did not affect their statistical significance (OR = 1.82; 95% CI = 1.40–2.38). These associations remained significant after further adjustment for waist circumference (model 4), with the exception of the obese non-sarcopenic group. Compared with the non-obese non-sarcopenic group, the ORs of hypertension in the non-obese sarcopenic, obese non-sarcopenic, and obese sarcopenic groups were 1.27 (95% CI, 1.02–1.60), 1.29 (0.98–1.67), and 1.65 (1.25–2.18) (*p* for trend <0.001). The risk of hypertension was significantly increased in subjects with sarcopenia, regardless of obesity. Controlling further for dietary sodium and potassium intakes (model 5) did not change the association between hypertension and sarcopenia.

**Table 3:** Unadjusted prevalence, adjusted odd ratios (OR) and 95% confidence intervals (CIs) for hypertension according the 4 body composition categories.

Hypertension	Non-obese	Non-obese	Obese	Obese	P for trend
	Non-sarcopenia	Sarcopenia	Non-sarcopenia	Sarcopenia	
Prevalence (%)	49.7 (1.4)	60.9 (2.0)	66.2 (2.2)	74.7 (1.5)	<0.001
Adjusted					
Model 1	1 (ref.)	1.50 (1.23, 1.84)	2.08 (1.68, 2.57)	3.00 (2.48, 3.63)	<0.001
Model 2	1 (ref.)	1.35 (1.09, 1.67)	1.81 (1.44, 2.27)	2.52 (2.06, 3.09)	<0.001
Model 3	1 (ref.)	1.35 (1.09, 1.69)	1.38 (1.06, 1.79)	1.82 (1.40, 2.38)	<0.001
Model 4	1 (ref.)	1.27 (1.02, 1.60)	1.29 (0.98, 1.67)	1.65 (1.25, 2.18)	<0.001
Model 5	1 (ref.)	1.33 (1.06, 1.67)	1.33 (0.99, 1.80)	1.89 (1.42, 2.51)	<0.001

Model 1 is adjusted for age, sex, regular activity, current smoking and alcohol use.

Model 2 is adjusted for age, sex, regular activity, current smoking, alcohol use, previous CHD, stroke and metabolic risk factors (fasting glucose, triglycerides, HDL cholesterol).

Model 3 is adjusted for age, sex, regular activity, current smoking, alcohol use, previous CHD, stroke, metabolic risk factors (fasting glucose, triglycerides, HDL cholesterol) and body weight.

Model 4 is adjusted for all variables in model 3 plus waist circumference.

Model 5 is adjusted for all variables in model 3 plus dietary sodium & potassium intake.

We performed an additional analysis to examine the independent effect of ASM (kg) on hypertension. In this analysis, we adjusted for age, sex, regular activity, current smoking, alcohol use, previous CHD, stroke, metabolic risk factors (fasting glucose, triglycerides, HDL cholesterol). The results of the logistic regression in which both ASM and BMI were used as continuous variables to predict the risk of hypertension are as follows: the adjusted OR for having hypertension was 0.94 (95% CI = 0.91–0.98) for ASM and 1.18 (95% CI = 1.12–1.24) for BMI (Table S2 in File S1). For every 1.0 kg increase in ASM, the odds of hypertension decreased by 6%, whereas for every 1.0 kg/m^2^ increase in BMI, the same odds increased by 18% after adjusting the effects of other covariates.

We performed an additional analysis to examine the independent effect of ASM (kg) on BP levels. Multiple linear regression analysis revealed that ASM (kg) was independently related to SBP (P = 0.024), not related to DBP (P = 0.525; Table S3 in File S1). It also revealed that age, current smoking, and heavy alcohol drinking were independently related to systolic/diastolic BP levels.

### Subgroup Analyses

We performed the analyses separately for men and women. The associations between sarcopenic obesity and hypertension were consistent in men and women (Table S4 in File S1). Among the men (*n* = 2,099), the age and lifestyle factors (regular activity, current smoking and alcohol use)–adjusted ORs for having hypertension were 1.69 (95% CI, 1.28–2.22) in subjects in the non-obese sarcopenia group, 2.08 (1.44–3.01) in the obese non-sarcopenia group and 2.90 (2.14–3.92) in the obese sarcopenia group, compared with the non-obese non-sarcopenia group (*p* for trend <0.001). Among the women (*n* = 2,747), the adjusted ORs for having hypertension were 1.39 (95% CI, 1.05–1.84) in subjects in the non-obese sarcopenia group, 2.12 (1.62–2.76) in the obese non-sarcopenia group and 3.07 (2.35–4.00) in the obese sarcopenia group, compared with the non-obese non-sarcopenia group (*p* for trend <0.001).

We performed the analyses separately in subjects with and without DM (Table S4 in File S1). Among the subjects without DM (*n* = 3,970), the various confounding factors (age, sex, regular activity, current smoking and alcohol use) adjusted ORs for having hypertension were 1.53 (95% CI, 1.21–1.93) in subjects in the non-obese sarcopenia group, 1.96 (1.52–2.53) in the obese non-sarcopenia group and 2.72 (2.18–3.38) in the obese sarcopenia group, compared with the non-obese non-sarcopenia group (*p* for trend <0.001). When analyses were confined to the subjects with diabetes mellitus (*n* = 876), the results from various covariates adjusted analysis were more robust than the previous findings (OR = 1.59, 95% CI = 1.01–2.52 for non-obese sarcopenia; OR = 2.54, 95% CI = 1.50–4.31 for obese non-sarcopenia; and OR = 3.95, 95% CI = 2.51–6.20 for obese sarcopenia).

We performed a sensitivity analysis with a BMI of 28 kg/m^2^ as a threshold to define obesity (Table S5 in File S1). The associations between sarcopenic obesity and hypertension were consistent after changing the threshold for obesity. The results were not quite different from those analyzed previously. Compared with the non-obese non-sarcopenic group, ORs of hypertension in the non-obese sarcopenic, obese non-sarcopenic, and obese sarcopenic groups were 1.69 (95% CI, 1.43–2.00), 2.37 (1.41–3.98), and 3.69 (2.67–5.09) (P for trend <0.001), respectively, after adjusting for the various confounding factors (age, sex, regular activity, current smoking and alcohol use).

## Discussion

We compared the prevalence and risk of hypertension according to the presence of sarcopenia in the non-obese and obese groups. We found that subjects with sarcopenia had a higher prevalence of hypertension than subjects without sarcopenia regardless of whether they were in the obese or non-obese group. Furthermore, sarcopenia was independently associated with hypertension after adjustment for potential confounders, particularly waist circumference.

Interestingly, we found a strong association between hypertension and sarcopenia in the participants with diabetes mellitus. Patients with diabetes mellitus were more likely to develop hypertension; the incidence of hypertension was twofold higher in subjects with diabetes mellitus relative to similarly aged individuals without diabetes mellitus [17]. In our study, the prevalence of hypertension among elderly Korean adults with diabetes was 70.3%. The risk of hypertension was fourfold higher in diabetic patients with obese sarcopenia relative to patients without. Hypertension should be screened more intensively in diabetic patients with obese sarcopenia due to their higher prevalence of hypertension. Prevention and timely treatment of sarcopenia (such as a diet and exercise programs aimed at increasing muscle mass and decreasing fat mass) may represent a valuable adjunctive therapy in diabetic patients to improve their hypertension management.

Our data demonstrated a link between sarcopenia and hypertension. Four possible mechanisms may explain how ageing muscle influences development of hypertension. First, muscle loss represents a decrease in the mass of insulin-responsive target tissue. This promotes insulin resistance, and thus obesity, metabolic syndrome and hypertension [18]. In our study, HOMA-IR was significantly higher in subjects with sarcopenia than in subjects without. Second, inflammation may also be a potential explanation for the association of sarcopenia with hypertension. In our study, WBC counts were significantly higher in patients with sarcopenia than in patients without. Third, subjects with sarcopenia commonly experience functional impairment and physical disability, which causes a reduction in muscle contraction-induced factors having an anti-inflammatory effect, also known as myokines [19]. The relative paucity of myokines in sarcopenia may increase the risk of CVD including hypertension [20], [21]. Fourth, alterations within the renin-angiotensin-aldosterone system (RAAS) may contribute to the development of sarcopenia and hypertension. Mineralocorticoid receptors’ activation in heart failure contributes to the progressive loss of cardiac myocytes by apoptosis [22]. Myocyte apoptosis also occurs in the skeletal muscles of patients with congestive heart failure (CHF) in a phenomenon known as ‘cardiac cachexia’, which in turn can lead to muscle wasting, weakness and reduced exercise tolerance in a process similar to sarcopenia [23]. The plasma concentration of aldosterone in CHF patients with cachexia is threefold greater than that in age-matched non-cachectic patients without heart failure [24].

A Japanese study demonstrated that sarcopenia was significantly associated with greater arterial stiffness, particularly in females [25]. Arterial stiffness is associated with a higher risk of newly developed high BP, also known as hypertension. Leg muscle mass was a more important determinant of central arterial stiffness than fat mass [3]. As muscle mass increases, so does the requirement for blood supply, resulting in a higher cardiac output, stroke volume and size adaptation of the arteries. This is demonstrated by the larger diameter and distension of both femoral and brachial arteries in people with more leg lean mass [3]. Larger leg lean mass was the most important determinant of lower arterial stiffness. In our study, ASM/Wt was negatively correlated with SBP and DBP. We demonstrated that sarcopenia was a determinant of BP in older Koreans.

Since BMI and body weight have been shown positively associated with BP levels, we performed an additional analysis to examine the independent effect of ASM (kg) on BP levels.

Multiple linear regression analysis revealed that ASM (kg) was independently related to SBP, not related to DBP. It also revealed that age, current smoking, and heavy alcohol drinking were independently related to systolic/diastolic BP levels. Current smokers tended to have lower BPs than never or past smokers, significantly so for DBP. Paradoxically, several epidemiological studies have found that BP levels among cigarette smokers were the same as or lower than those of nonsmokers [26]. Smoking is strongly associated with alcohol intake. Heavy alcohol intake appears to affect skeletal muscle severely, promoting its damage and wasting [27]. Therefore, heavy alcohol drinking and smoking must be taken into account when assessing the relationship between sarcopenia and hypertension. In our study, we found that the association between ASM and blood pressure was independent of these confounding factors.

The major strength of this study is that we used data from a large representative sample of the elderly general population in Korea. Moreover, skeletal muscle mass was directly measured by DXA. Our study also had several limitations. First, we used a cross-sectional design, which limits the ability to detect causal relationships. Therefore, a cause-and-effect relationship between sarcopenia and hypertension cannot be inferred. Second, the subjects were older Koreans. Therefore, our findings cannot be extrapolated to other racial groups. Third, hypertension was defined based on blood pressure measurement only on one occasion. Therefore, white-coat effect and random variability in BP might influence the validity and reliability in diagnosing hypertension. This limitation may result in either an underestimation or overestimation of the association between sarcopenia and hypertension. Fourth, there was preference for zero end-digit in the BP measurement. End digit preference is an indicator of low-quality BP measurement. This might influence the diagnosis of hypertension.

In conclusion, body composition beyond BMI has a considerable impact on hypertension in elderly Koreans. Subjects with obese sarcopenia appear to have a greater risk of hypertension than simply obese or sarcopenia subjects. Considering that the Korean population is becoming older and more obese, body-composition-specific public health interventions are needed for the prevention and treatment of hypertension.

## Supporting Information

File S1 **Table S1, Distribution of the last digits recorded for systolic and diastolic blood pressure values. Table S2, Multiple logistic regression analysis for hypertension as a dependent variable. Table S3, Multiple linear regression analysis of systolic blood pressure (SBP)/diastolic blood pressure (DBP) with ASM and other confounding factors. Table S4, Unadjusted prevalence, adjusted Odd ratios (OR), and 95% confidence intervals (CIs) for hypertension according the 4 body composition categories in men and women (upper part)/non-diabetes mellitus and diabetes mellitus subjects (lower part). Table S5, Unadjusted prevalence, adjusted Odd ratios (OR), and 95% confidence intervals (CIs) for hypertension according the 4 body composition categories, defined obesity as BMI ≥28 kg/m^2^.**
(DOC)
