# Supplementary material for: Sarcopenia as a Determinant of Blood Pressure in Older Koreans: Findings from the Korea National Health and Nutrition Examination Surveys (KNHANES) 2008–2010
Source: PLoS One. 2014 Jan 29;9(1):e86902. doi: 10.1371/journal.pone.0086902 (PMC3906091; doi:10.1371/journal.pone.0086902)
Supplement: File S1 — Table S1, Distribution of the last digits recorded for systolic and diastolic blood pressure values. Table S2, Multiple logistic regression analysis for hypertension as a dependent variable. Table S3, Multiple linear regression analysis of systolic blood pressure (SBP)/diastolic blood pressure (DBP) with ASM and other confounding factors. Table S4, Unadjusted prevalence, adjusted Odd ratios (OR), and 95% confidence intervals (CIs) for hypertension according the 4 body composition categories in men and women (upper part)/non-diabetes mellitus and diabetes mellitus subjects (lower part). Table S5, Unadjusted prevalence, adjusted Odd ratios (OR), and 95% confidence intervals (CIs) for hypertension according the 4 body composition categories, defined obesity as BMI ≥28 kg/m2. (DOC) [file pone.0086902.s001.doc]

Table S1. Distribution of the last digits recorded for systolic and diastolic blood pressure values

| Last Digit | 2^nd^ SBP | 2^nd^ DBP | 3^rd^ SBP | 3^rd^ DBP |
| --- | --- | --- | --- | --- |
| 0 | 53.9 | 37.2 | 37.9 | 44.5 |
| 2 | 10.5 | 14.1 | 14.7 | 14.9 |
| 4 | 16.9 | 19.3 | 18.7 | 17.3 |
| 6 | 7.0 | 13.8 | 13.2 | 9.6 |
| 8 | 11.7 | 15.6 | 15.5 | 13.7 |
| Total | 100 | 100 | 100 | 100 |

Table S2. Multiple logistic regression analysis for hypertension as a dependent variable

|  | Hypertension |  |
| --- | --- | --- |
|  | OR (95% CI) | P-value |
| ASM (kg) | 0.94 (0.91, 0.98) | 0.003 |
| BMI (kg/m^2^) | 1.18 (1.12, 1.24) | <0.001 |
| Age (years) | 1.04 (1.03, 1.06) | <0.001 |
| Sex (Female vs. male) | 0.77 (0.55, 1.07) | 0.119 |
| Regular activity (yes vs. no) | 0.94 (0.79, 1.12) | 0.512 |
| Current smoking (yes vs. no) | 0.73 (0.59, 0.91) | 0.006 |
| Alcohol use (heavy drinker vs. non-drinker)^a^ | 1.66 (1.16, 2.35) | 0.002 |
| Diabetes mellitus (yes vs. no) | 1.44 (1.16, 1.77) | <0.001 |
| Dyslipidemia (yes vs. no) | 1.44 (1.16, 1.78) | <0.001 |
| Previous CHD (yes vs. no) | 1.27 (0.86, 1.88) | 0.225 |
| Previous Stroke (yes vs. no) | 1.80 (1.05, 3.09) | 0.031 |

^a^ Heavy alcohol drinking was defined as having more than 30g alcohol per day.

Table S3. Multiple linear regression analysis of systolic blood pressure (SBP) / diastolic blood pressure (DBP) with ASM and other confounding factors

|  | SBP | | DBP | |
| --- | --- | --- | --- | --- |
|  | β | P-value | β | P-value |
| ASM (kg) | –0.370 | 0.024 | 0.059 | 0.525 |
| BMI (kg/m^2^) | 0.677 | 0.001 | 0.650 | <0.001 |
| Age (years) | 0.463 | <0.001 | –0.177 | <0.001 |
| Sex (Female vs. male) | –0.209 | 0.871 | –0.083 | 0.909 |
| Regular activity (yes vs. no) | 0.348 | 0.652 | –0.444 | 0.289 |
| Current smoking (yes vs. no) | –1.957 | 0.055 | –2.136 | <0.001 |
| Alcohol use (heavy drinker vs. non-drinker)^a^ | 5.552 | <0.001 | 4.022 | <0.001 |
| Diabetes mellitus (yes vs. no) | 1.321 | 0.085 | –1.677 | <0.001 |
| Dyslipidemia (yes vs. no) | 1.164 | 0.114 | 1.054 | 0.013 |
| Previous CHD (yes vs. no) | –1.561 | 0.252 | –1.647 | 0.133 |
| Previous Stroke (yes vs. no) | 0.572 | 0.727 | 1.384 | 0.170 |

Table S4. Unadjusted prevalence, adjusted Odd ratios (OR), and 95% confidence intervals (CIs) for hypertension according the 4 body composition categories in men and women (upper part) / non-diabetes mellitus and diabetes mellitus subjects (lower part)

|  | | Non-obese  Non-sarcopenia | Non-obese  Sarcopenia | Obese  Non-sarcopenia | Obese  Sarcopenia | P for trend |
| --- | --- | --- | --- | --- | --- | --- |
| **Men (n= 2,099)** | |  |  |  |  |  |
|  | Unadjusted prevalence | 45.7 (2.0) | 60.0 (2.9) | 62.6 (3.8) | 70.8 (2.7) | <0.001 |
|  | Adjusted OR* | 1(ref.) | 1.69 (1.28, 2.22) | 2.08 (1.44, 3.01) | 2.90 (2.14, 3.92) | <0.001 |
| **Women (n= 2,747)** | | |  |  |  |  |
|  | Unadjusted prevalence | 53.0 (1.8) | 61.8 (2.8) | 68.2 (2.6) | 76.9 (1.9) | <0.001 |
|  | Adjusted OR* | 1(ref.) | 1.39 (1.05, 1.84) | 2.12 (1.62, 2.76) | 3.07 (2.35, 4.00) | <0.001 |
| **Non-DM (n= 3,970)** | |  |  |  |  |  |
|  | Unadjusted prevalence | 48.0 (1.5) | 60.3 (2.4) | 63.5 (2.6) | 71.4 (1.9) | <0.001 |
|  | Adjusted OR* | 1(ref.) | 1.53 (1.21, 1.93) | 1.96 (1.52, 2.53) | 2.72 (2.18, 3.38) | <0.001 |
| **DM (n= 876)** | |  |  |  |  |  |
|  | Unadjusted prevalence | 57.9 (3.4) | 67.3 (4.0) | 75.0 (4.2) | 83.8 (2.5) | <0.001 |
|  | Adjusted OR* | 1(ref.) | 1.59 (1.01, 2.52) | 2.54 (1.50, 4.31) | 3.95 (2.51, 6.20) | <0.001 |

*adjusting for age, sex, regular activity, current smoking and alcohol use.

Table S5. Unadjusted prevalence, adjusted Odd ratios (OR), and 95% confidence intervals (CIs) for hypertension according the 4 body composition categories, defined obesity as BMI ≥ 28 kg/m^2^

| Hypertension | Non-obese  Non-sarcopenia | Non-obese  Sarcopenia | Obese  Non-sarcopenia | Obese  Sarcopenia | P for trend |
| --- | --- | --- | --- | --- | --- |
| Prevalence (%) | 52.6 (1.2) | 65.4 (1.5) | 70.1 (5.4) | 81.9 (2.4) | <0.001 |
| Adjusted |  |  |  |  |  |
| Model 1 | 1 (ref.) | 1.69 (1.43, 2.00) | 2.37 (1.41, 3.98) | 3.69 (2.67, 5.09) | <0.001 |
| Model 2 | 1 (ref.) | 1.59 (1.34, 1.89) | 2.43 (1.40, 4.22) | 3.38 (2.41, 4.73) | <0.001 |
| Model 3 | 1 (ref.) | 1.35 (1.13, 1.61) | 1.33 (0.72, 2.44) | 1.74 (1.19, 2.54) | <0.001 |
| Model 4 | 1 (ref.) | 1.27 (1.06, 1.53) | 1.26 (0.68, 2.34) | 1.63 (1.09, 2.42) | <0.001 |
| Model 5 | 1 (ref.) | 1.37 (1.13, 1.65) | 1.11 (0.58, 2.14) | 1.80 (1.19, 2.73) | <0.001 |

Model 1 is adjusted for age, sex, regular activity, current smoking and alcohol use.

Model 2 is adjusted for age, sex, regular activity, current smoking, alcohol use, previous CHD, stroke and metabolic risk factors (fasting glucose, triglycerides, HDL cholesterol).

Model 3 is adjusted for age, sex, regular activity, current smoking, alcohol use, previous CHD, stroke, metabolic risk factors (fasting glucose, triglycerides, HDL cholesterol) and body weight.

Model 4 is adjusted for all variables in model 3 plus waist circumference.

Model 5 is adjusted for all variables in model 3 plus dietary sodium & potassium intake.
